# Use of Readily Accessible Inflammatory Markers to Predict Diabetic Kidney Disease

**DOI:** 10.3389/fendo.2018.00225

**Published:** 2018-05-22

**Authors:** Lauren Winter, Lydia A. Wong, George Jerums, Jas-mine Seah, Michele Clarke, Sih Min Tan, Melinda T. Coughlan, Richard J. MacIsaac, Elif I. Ekinci

**Affiliations:** ^1^Endocrine Centre of Excellence, Austin Health, Melbourne, VIC, Australia; ^2^Department of Medicine, Austin Health, University of Melbourne, Melbourne, VIC, Australia; ^3^Department of Diabetes, Central Clinical School, Monash University, Melbourne, VIC, Australia; ^4^Department of Endocrinology and Diabetes, St Vincent’s Health, Melbourne, VIC, Australia; ^5^Department of Medicine, St Vincent’s Health, University of Melbourne, Melbourne, VIC, Australia

**Keywords:** diabetic kidney disease, neutrophil–lymphocyte ratio, inflammation, neutrophils, lymphocytes, diabetic nephropathy, atherosclerosis

## Abstract

Diabetic kidney disease is a common complication of type 1 and type 2 diabetes and is the primary cause of end-stage renal disease in developed countries. Early detection of diabetic kidney disease will facilitate early intervention aimed at reducing the rate of progression to end-stage renal disease. Diabetic kidney disease has been traditionally classified based on the presence of albuminuria. More recently estimated glomerular filtration rate has also been incorporated into the staging of diabetic kidney disease. While albuminuric diabetic kidney disease is well described, the phenotype of non-albuminuric diabetic kidney disease is now widely accepted. An association between markers of inflammation and diabetic kidney disease has previously been demonstrated. Effector molecules of the innate immune system including C-reactive protein, interleukin-6, and tumor necrosis factor-α are increased in patients with diabetic kidney disease. Furthermore, renal infiltration of neutrophils, macrophages, and lymphocytes are observed in renal biopsies of patients with diabetic kidney disease. Similarly high serum neutrophil and low serum lymphocyte counts have been shown to be associated with diabetic kidney disease. The neutrophil–lymphocyte ratio is considered a robust measure of systemic inflammation and is associated with the presence of inflammatory conditions including the metabolic syndrome and insulin resistance. Cross-sectional studies have demonstrated a link between high levels of the above inflammatory biomarkers and diabetic kidney disease. Further longitudinal studies will be required to determine if these readily available inflammatory biomarkers can accurately predict the presence and prognosis of diabetic kidney disease, above and beyond albuminuria, and estimated glomerular filtration rate.

## Introduction

In patients with type 1 and type 2 diabetes mellitus, methods to assist in the early identification of kidney disease are a priority so that measures to prevent its progression can be put in place. Newer medications to prevent diabetic kidney disease are increasingly becoming available that may offer benefits in addition to good glycemic and blood pressure control and inhibition of the renin–angiotensin system. These newer medications include sodium glucose co-transporter-linked blockers (1, 2), and glucagon-like peptide-1 analogs (3, 4) are already being used in clinical practice, and the anticipated release of novel antifibrotic agents and anti-monocyte chemoattractant protein-1 agents is likely to help prevent the onset of diabetic kidney disease (5).

Currently, diabetic kidney disease accounts for the majority of cases of end-stage renal disease in developed countries (6) and is of increasing concern in developing countries (7). It is estimated that 40% of individuals with either type 1 or type 2 diabetes will go on to develop diabetic kidney disease (8). Rates of type 2 diabetes alone are estimated to double within the next 25 years to 352 million worldwide (7). Increasing numbers of patients with diabetes and subsequently diabetic kidney disease will place a significant financial burden on the healthcare system (9).

The full pathogenesis of diabetic kidney disease is still being elicited (10); however, early detection and subsequent intervention may slow rates of disease progression (11). Current diagnosis of diabetic kidney disease is based on the presence of reduced estimated glomerular filtration rate and/or an increased urinary albumin excretion in absence of other renal disease. The phenotype of normoalbuminuric renal insufficiency is also becoming increasingly recognized (12). The limitations of using albuminuria alone as a marker of diabetic kidney disease are well recognized, emphasizing the importance of developing novel, cheap, and accessible ways to detect early renal disease which will be useful in the context of the increasing burden of diabetes (13, 14). Improving our understanding of the pathogenesis of diabetic kidney disease is likely to assist in the management of patients (15).

Activation of immune and inflammatory pathways is increasingly recognized as key mediators for the development and progression of renal damage in diabetes with diabetic kidney disease (16). In this review, we focus on the potential role of readily accessible indicators of activation of the innate and adaptive immune system as markers of diabetic kidney disease. In particular, we review evidence for the immune cells neutrophils, macrophages, and monocytes which reflect innate immunity and lymphocytes which reflect adaptive immunity as indicators for the presence of diabetic kidney disease.

## Discussion

### Overview of the Immune System

**Figure 1:**
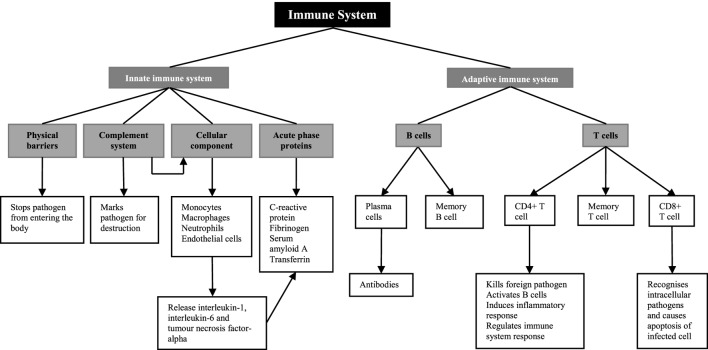
Overview of the immune system.

The role of the immune system is to defend the human body against injury from foreign pathogens, toxins, and allergens (17) (Figure 1). There are two components of the immune system; the innate immune system and the adaptive immune system (18). The innate immune system provides the first line of defense when a foreign molecule is detected. It involves physical barriers such as the epidermis (19), the complement system (19), a cellular component including both neutrophils and macrophages and the acute-phase response (18). Physical barriers act to stop foreign pathogens from entering the body (17). These include the epithelial cell layers that form tight cell–cell contacts, the secreted mucus layer lining the epithelium of the respiratory, gastrointestinal, and genitourinary tracts, and the epithelial cilia that help to move particles out of the body (17). The complement system, which involves plasma and cell surface proteins, marks foreign pathogens for destruction (17). In addition to this, it activates the cellular component of the innate immune system (17). This includes neutrophils, macrophages, and CD4+ T cells, members of the adaptive immune system (17). On and in, these cells are a number of pattern recognition receptors, including toll-like receptors and the receptor for advanced glycation end products that recognize danger-associated molecular patterns. In diabetic kidneys, toll-like receptors 1 and 2 as well as the receptor for advanced glycation end products are thought to be the main receptors for danger-associated molecular patterns in promoting immune-mediated injury (16). Increased expression of these receptors in diabetic kidney disease leads to the increased activation of myeloid differentiation primary response 88 or nuclear factor-κB and production of pro-inflammatory cytokines (16), which are the main mediators of inflammation and the acute-phase response (20).

Pro-inflammatory cytokines, including interleukin-6 and tumor necrosis factor-alpha released from macrophages, monocytes, and endothelial cells stimulate the liver to synthesize acute-phase proteins (21), such as C-reactive protein, fibrinogen, and complement (20). C-reactive protein acts as opsonin for bacteria, parasites, and immune complexes and can activate the classical pathway of complement (22, 23). Furthermore, it induces the release of pro-inflammatory cytokines and tissue factors in monocytes (24, 25). Increase in the plasma concentration of complement cascade components can lead to the accumulation of neutrophils, macrophages, and plasma proteins, participating in the killing of infectious agents, the clearance of foreign and host cellular debris, and the repair of damaged tissue (23). Fibrinogen, which is a component of the coagulation cascade, plays an essential role in promoting wound healing (23).

While the innate immune system is the host’s non-specific resistance to pathogens and is poised to act rapidly, the adaptive immune system acts in a highly specific nature involving antigen specificity and immunologic memory (26). The adaptive immune system involves lymphocytes known as T cells and B cells, and both express highly specific antigen receptors on their surface (27). T cells are important mediators of the cell-mediated immune response, whereas B cells are critical for the humoral immune response.

There are two main types of T cells; CD4+ and CD8+ T cells (27). CD4+ T cells function as both helper and regulatory cells and are activated by antigen presenting cells such as macrophages and influence the nature of the immune response (27). Cytotoxic CD8+ T cells recognize intracellular pathogens and activate apoptosis of the infected cell (27). B cells are defined by their production of immunoglobulin, or antibody, against a specific antigen, also known as humoral immunity (17). Once a B cell encounters an antigen and is activated, it will become either a memory cell to be activated once again in the future or it will become a plasma cell, producing large amounts of antibody (27).

### Inflammation in Type 2 Diabetes Mellitus

Chronic systemic inflammation has been hypothesized to contribute to the development of type 2 diabetes (28) as well as its complications (29). Activation of inflammatory pathways is common to type 2 diabetes, atherosclerosis, and cardiovascular disease (30). Furthermore, the metabolic syndrome, which includes insulin resistance, type 2 diabetes, hypertension, dyslipidemia, and central obesity (31), is a condition of chronic low-grade inflammation (32). Importantly, anti-inflammatory therapies such as canakinumab, a monoclonal antibody against interleukin-1β, has been shown to reduce the risk of cardiovascular disease by reducing inflammation without lowering baseline lipid levels (33).

#### Acute-Phase Proteins and Pro-Inflammatory Cytokines in the Development of Type 2 Diabetes Mellitus

It is thought that innate immune system activation may play a role in the development and progression of type 2 diabetes and its complications (18). There have been a small number of cross-sectional analyses performed regarding the relationship between circulating C-reactive protein, interleukin-6, fibrinogen, and type 2 diabetes (34–36) (Table 1). There have been many more longitudinal studies examining whether baseline measurements can predict the development of type 2 diabetes within a given follow-up period (37–50) (Table 2). The majority of these studies showed a positive relationship between C-reactive protein and the risk of developing type 2 diabetes. The strength of these associations was often lessened when adjusting for measures of adiposity, some to the point of losing significance. In general, these studies reported no significance between C-reactive protein and the risk of developing type 2 diabetes after adjusting for body mass index (51), waist–hip ratio in addition to body mass index (50) and without adjusting for any patient characteristics (52). Overall, however, these results suggest that having markers of chronic low-grade inflammation is indeed associated with an increased risk of developing type 2 diabetes.

**Table 1:** Cross-sectional studies of C-reactive protein, interleukin-6, and tumor necrosis factor-α in type 2 diabetes.

Reference	Groups	Endpoint	Findings	*p* Value
Ford (34)	Impaired fasting glucose (*n* = 685) vs normal fasting glucose (*n* = 7,952)	Odds ratio for elevated C-reactive protein	0.99 (0.72–1.37)^a^	Not given
	Newly diagnosed diabetes (*n* = 315) vs normal fasting glucose (*n* = 7,952)	Odds ratio for elevated C-reactive protein	1.84 (1.25–2.71)^a^	Not given
	Previously diagnosed diabetes (*n* = 1,367) vs normal fasting glucose (*n* = 7,952)	Odds ratio for elevated C-reactive protein	1.59 (1.25–2.01)^a^	Not given

Muller et al. (35)	Impaired glucose tolerance (*n* = 80) vs controls (*n* = 77)	Median interleukin-6	1.8 vs 0.8 pg/ml	*p* < 0.0001
	Type 2 diabetes (*n* = 152) vs controls (*n* = 77)	Median interleukin-6	2.5 vs 0.8 pg/ml	*p* < 0.0001

Pitsavos et al. (36)	Type 2 diabetes (*n* = 210) vs controls (*n* = 2,338)	Mean C-reactive protein	3.08 ± 3.36 vs 1.96 ± 2.83 mg/l	*p* < 0.001
	Type 2 diabetes (*n* = 210) vs controls (*n* = 2,338)	Mean interleukin-6	1.72 ± 0.47 vs 1.41 ± 0.52 pg/ml	*p* < 0.001
	Type 2 diabetes (*n* = 210) vs controls (*n* = 2,338)	Mean tumor necrosis factor-α	9.50 ± 7.41 vs 5.95 ± 4.78 pg/ml	*p* < 0.001

*^a^95% Confidence interval*.

**Table 2:** Longitudinal studies of C-reactive protein, interleukin-6, tumor necrosis factor-α, fibrinogen, and plasminogen activator inhibitor-1 in type 2 diabetes.

Reference	Follow-up period (years)	Groups	Endpoint	Findings	*p* Value
Pradhan et al. (37)	4	Type 2 diabetes (*n* = 188) vs matched controls (*n* = 362)	Baseline median interleukin-6	2.0 (1.43, 2.78) vs 1.38 (0.91, 2.05) pg/ml^b^	*p* < 0.001
		Type 2 diabetes (*n* = 188) vs matched controls (*n* = 362)	Baseline median C-reactive protein	0.69 (0.42, 1.00) vs 0.26 (0.10, 0.61) mg/dl^b^	*p* < 0.001
		Baseline interleukin-6 quartiles	Relative risk of type 2 diabetes for quartile 1, 2, 3, and 4	1.0, 2.5 (1.1, 5.6), 4.1 (2.0, 8.4), and 7.5 (3.7, 15.4)^b^	*p* < 0.001
		Baseline C-reactive protein quartiles	Relative risk of type 2 diabetes for quartile 1, 2, 3, and 4	1.0, 2.2 (0.8, 6.0), 8.7 (3.6, 21.0), and 15.7 (6.5, 37.9)^b^	*p* < 0.001

Hu et al. (38)	10	Type 2 diabetes (*n* = 737) vs matched controls (*n* = 785)	Baseline median C-reactive protein	0.36 vs 0.16 mg/dl	*p* < 0.001
		Type 2 diabetes (*n* = 737) vs matched controls (*n* = 785)	Baseline median interleukin-6	2.38 vs 1.84 pg/ml	*p* < 0.001
		Type 2 diabetes (*n* = 737) vs matched controls (*n* = 785)	Baseline median tumor necrosis factor-α receptor 2	2,646.5 vs 2,383.8 pg/ml	*p* < 0.001
		Baseline C-reactive protein quintiles	Odds ratio for type 2 diabetes: highest quintile vs lowest quintile	4.36 (2.80–6.80)^a^	*p* < 0.001
		Baseline interleukin-6 protein quintiles	Odds ratio for type 2 diabetes: highest quintile vs lowest quintile	1.91 (1.27–2.86)^a^	*p* < 0.001
		Baseline tumor necrosis factor-α receptor 2 protein quintiles	Odds ratio for type 2 diabetes: highest quintile vs lowest quintile	1.64 (1.10–2.45)^a^	*p* < 0.001

Freeman et al. (39)	5	Type 2 diabetes (*n* = 127) vs non diabetics (5,118) after follow-up	Hazard ratio of baseline C-reactive protein to predict type 2 diabetes	1.55 (1.32–1.82)^a^	*p* < 0.0001
		Baseline C-reactive protein quintiles	Hazards ratio to predict type 2 diabetes: highest quintile vs lowest quintile	6.13 (2.76–13.60)^a^	*p* < 0.0001

Laaksonen et al. (40)	11	Baseline C-reactive protein ≥ 3.0 mg/l (*n* = 348) vs < 1.0 mg/l (*n* = 106)	Odds ratio of developing type 2 diabetes	4.11 (2.11–7.98)^a^	*p* < 0.001

Morimoto et al. (41)	5	Type 2 diabetes (*n* = 316) vs controls (*n* = 7,076) after follow-up	Odds ratio of 2.9-fold increase in baseline C-reactive protein	1.8 (1.03–1.34)^a^	*p* = 0.018
		Type 2 diabetes (*n* = 316) vs controls (*n* = 7,076) after follow-up	Odds ratio of 2.9-fold increase in C-reactive protein from baseline	1.21 (1.03–1.41)^a^	*p* = 0.018

Han et al. (42)	6	Baseline C-reactive protein tertile (total *n* = 729)	Relative risk of developing type 2 diabetes: highest tertile vs lowest tertile	5.4 (2.2–13.4)^a^	*p* < 0.001

Thorand et al. (43)	10.8	Baseline C-reactive protein women type 2 diabetes (*n* = 222)	Hazard ratio to predict type 2 diabetes: highest tertile vs lowest tertile	7.60 (4.43–13.04)^a^	*p* < 0.001
		Baseline C-reactive protein men type 2 diabetes (*n* = 305)	Hazard ratio to predict type 2 diabetes: highest tertile vs lowest tertile	1.84 (1.27–2.67)^a^	*p* < 0.001

Nakanishi et al. (44)	6.54	Baseline C-reactive protein women type 2 diabetes (*n* = 551)	Hazard ratio to predict type 2 diabetes: highest tertile vs lowest tertile	3.11 (1.25–7.75)^a^	*p* = 0.036
		Baseline C-reactive protein men type 2 diabetes (*n* = 396)	Hazard ratio to predict type 2 diabetes: highest tertile vs lowest tertile	2.84 (1.09–7.39)^a^	*p* = 0.035

Barzilay et al. (45)	3–4	Type 2 diabetes (*n* = 52) vs non diabetic (*n* = 4,436) after follow-up	Baseline median C-reactive protein	2.94 (1.44, 7.0) vs 1.62 (0.81, 2.81) mg/l^b^	*p* < 0.001
		Baseline C-reactive protein (*n* = 4,481)	Odds ratio of type 2 diabetes: 75th percentile vs 25th percentile	2.03 (1.44–2/86)^a^	not given

Festa et al. (46)	5.2	Type 2 diabetes (*n* = 144) vs non diabetic (*n* = 903) after follow-up	Baseline mean C-reactive protein	2.40 (1.29, 5.87) vs 1.67 (0.75, 3.41) mg/l^b^	*p* = 0.0001
		Type 2 diabetes (*n* = 144) vs non diabetic (*n* = 903) after follow-up	Baseline mean fibrinogen	287.8 ± 58.8 vs 275.1 ± 56 mg/dl^c^	*p* = 0.013
		Type 2 diabetes (*n* = 144) vs non diabetic (*n* = 903) after follow-up	Baseline mean plasma plasminogen activator inhibitor-1	24 (15, 37.5) vs 16 (9, 27) ng/ml^b^	*p* = 0.0001
		Baseline C-reactive protein quartiles	Incidence of type 2 diabetes in each quartile 1, 2, 3, 4 after follow-up	6.9, 12.1, 16.2, and 19.9%	*p* = 0.001
		Baseline plasminogen activator inhibitor-1 quartiles	Incidence of type 2 diabetes in each quartile 1, 2, 3, 4 after follow-up	6.6, 10.4, 15.6, and 23.1%	*p* = 0.001

Spranger et al. (47)	2.3	Type 2 diabetes (*n* = 188) vs matched controls (*n* = 377)	Baseline mean C-reactive protein	4.14 ± 5.1 vs 2.45 ± 4.38 μg/ml^c^	*p* < 0.0001
		Type 2 diabetes (*n* = 188) vs matched controls (*n* = 377)	Baseline mean interleukin-6	2.45 ± 1.80 vs 1.67 ± 1.59 pg/ml^c^	*p* < 0.0001
		Type 2 diabetes (*n* = 188) vs matched controls (*n* = 377)	Baseline mean tumor necrosis factor-α	2.04 ± 1.51 vs 1.79 ± 1.28 pg/ml^c^	*p* = 0.0094
		Baseline interleukin-6	Odds ratio of type 2 diabetes: highest quartile vs lowest quartile	2.57 (1.24–5.47)^a^	not given

Doi et al. (48)	9	Baseline C-reactive protein men (*n* = 694)	Odds ratio of type 2 diabetes: highest tertile vs lowest tertile	2.63 (1.23–5.65)^a^	*p* = 0.014
		Baseline C-reactive protein women (*n* = 1,065)	Odds ratio of type 2 diabetes: highest tertile vs lowest tertile	2.25 (1.01–5.01)^a^	*p* = 0.049

Dehghan et al. (49)	9.8	Baseline C-reactive protein (*n* = 5,901)	Hazard ratio to predict type 2 diabetes for second, third, and fourth quartile compared with first	1.88 (1.42–2.48), 2.16 (1.64–2.84), and 2.83 (2.16–3.70)^a^	*p* < 0.001

Lee et al. (50)	3.7	Baseline C-reactive protein (*n* = 1,001)	Odds ratio of type 2 diabetes: highest tertile vs lowest tertile	1.49 (1.03–2.15)^a^	*p* = 0.03

*^a^95% Confidence interval*.

*^b^Interquartile range*.

*^c^SD*.

#### White Cell Count in the Development of Type 2 Diabetes Mellitus

Several studies have examined the relationship between total white cell count and type 2 diabetes. The white cell count includes neutrophils, lymphocytes, monocytes, eosinophils, and basophils (53). Both cross-sectional studies (54, 55) (Table 3) and longitudinal studies (56, 57) (Table 4) have assessed this relationship between leukocytes and type 2 diabetes. These studies suggest particular white cells may be implicated in the development of type 2 diabetes and diabetic kidney disease based on albuminuria status.

**Table 3:** Cross-sectional studies of the white cell count in type 2 diabetes.

Reference	Groups	Endpoint	Findings	*p* Value
Ohshita et al. (55)	Impaired glucose tolerance (*n* = 476) vs impaired fasting glucose (*n* = 290)	Mean white cell count	6,530 vs 6,210/mm^3^	*p* < 0.05

Chung et al. (53)	Normoalbuminuria (*n* = 888) vs microalbuminuria (*n* = 326) vs overt nephropathy (*n* = 266)	Mean white cell count	6,572 ± 1,647 vs 6,984 ± 1,662 vs 7,440 ± 1,769 × 10^9^/l^a^	*p* < 0.0001
	Normoalbuminuria (*n* = 888) vs microalbuminuria (*n* = 326) vs overt nephropathy (*n* = 266)	Mean neutrophil count	3,730 ± 1,283 vs 4,101 ± 1,432 vs 4,742 ± 1,590 × 10^9^/l^a^	*p* < 0.0001
	Normoalbuminuria (*n* = 888) vs microalbuminuria (*n* = 326) vs overt nephropathy (*n* = 266)	Mean lymphocyte count	2,222 ± 736 vs 2,206 ± 701 vs 1,937 ± 769 × 10^9^/l^a^	*p* < 0.001
	Normoalbuminuria (*n* = 888) vs microalbuminuria (*n* = 326) vs overt nephropathy (*n* = 266)	Mean monocyte count	440 ± 158 vs 482 ± 173 vs 553 ± 237 × 10^9^/l^a^	*p* < 0.0001

Cavalot et al. (54)	Microalbuminuria (*n* = 140) vs normoalbuminuria (*n* = 476)	Mean white cell count	7,539 ± 1,882 vs 6,882 ± 1,713 μ/l^a^	*p* = 0.0051
	Macroalbuminuria (*n* = 43) vs normoalbuminuria (*n* = 476)	Mean white cell count	7,574 ± 1,981 vs 6,882 ± 1,713 μ/l^a^	*p* = 0.0143

*^a^SD*.

**Table 4:** Longitudinal studies of the white cell count in type 2 diabetes.

Reference	Follow-up period (years)	Groups	Endpoint	Findings	*p* Value
Schmidt et al. (58)	7	Baseline white cell count (*n* = 12,330)	Odds ratio highest quartile vs lowest quartile	1.9 (1.6–2.3)^a^	Not given
		Baseline neutrophil count (*n* = 12,330)	Odds ratio highest quartile vs lowest quartile	1.8 (1.5–2.3)^a^	Not given
		Baseline lymphocyte count (*n* = 12,330)	Odds ratio highest quartile vs lowest quartile	1.3 (1.1–1.7)^a^	Not given

Ford (56)	20	Baseline leukocyte count ≥ 9 × 10^9^/l vs <5.7 × 10^9^/l women (4,520)	Hazard ratio	1.68 (1.21–2.34)^a^	*p* = 0.002

Vozarova et al. (57)	5.5	Baseline white cell count (*n* = 352)	Relative hazard of 90th percentile vs 10th percentiles	2.7 (1.3–5.4)^a^	*p* = 0.007

*^a^95% Confidence interval*.

### Innate Immune System and the Development of Diabetic Kidney Disease

#### Acute-Phase Proteins and Pro-Inflammatory Cytokines in the Development of Diabetic Kidney Disease in Type 2 Diabetes Mellitus

Acute-phase proteins and pro-inflammatory cytokines have been implicated in the development of diabetic kidney disease. Albuminuria is often present in patients with diabetic kidney disease but there is also a subset of patients who develop diabetic kidney disease, as determined by declining glomerular filtration rate, without the presence of an elevated albumin excretion rate (59–61). Despite this, many studies continue to use urinary albumin excretion alone to determine the presence of diabetic kidney disease. In addition to this, microalbuminuria is thought to represent widespread endothelial dysfunction and vascular damage (62) and therefore may not always represent the presence of diabetic kidney disease (63). Microalbuminuria in type 2 diabetes is known to predict cardiovascular mortality (64) which may suggest a common underlying process such as inflammation (65).

Multiple studies have examined a relationship between microalbuminuria and acute-phase proteins, without necessarily using microalbuminuria to infer the presence of diabetic kidney disease. In a cross-sectional study of 467 patients and 1,014 controls, a significant positive correlation between microalbuminuria and both C-reactive protein and fibrinogen was demonstrated (66). In 64 patients, C-reactive protein and fibrinogen were significantly higher in those with microalbuminuria compared with those with normoalbuminuria (65). Elevated C-reactive protein and microalbuminuria at baseline resulted in an increased risk of death in 328 patients followed for 10 years (62). C-reactive protein was also found to be strongly associated with increasing albuminuria with annual follow-up.

**Figure 2:**
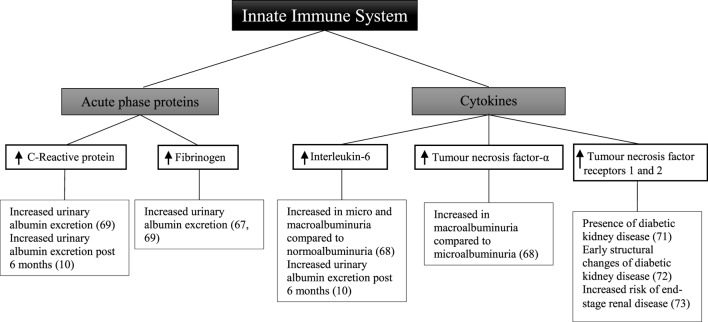
Inflammatory components of the innate immune system and diabetic kidney disease in type 2 diabetes.

Other studies have been designed to determine the relationship between circulating acute-phase proteins, pro-inflammatory cytokines, and diabetic kidney disease (Figure 2). In a small sample of 32 patients, there was a positive correlation between urinary albumin excretion and circulating fibrinogen (67). There was no significant relationship observed, however, with C-reactive protein and interleukin-6. In 151 patients compared with 80 matched controls, there was no significant relationship between interleukin-6 and albuminuria (68). There was, however, a significant difference in interleukin-6 between patients with either microalbuminuria or macroalbuminuria when compared with those with normoalbuminuria. Tumor necrosis factor-α levels were significantly different between patients with microalbuminuria and those with macroalbuminuria. Similarly circulating tumor necrosis factor-α was also significantly higher in all patients compared with controls.

A cross-sectional study of 74 patients with type 2 diabetes demonstrated that there was no significant relationship between fibrinogen, total leukocyte count, and glomerular filtration rate (69). Similarly in a subset of 66 patients, there was no association seen with C-reactive protein. Fibrinogen, total leukocyte count, and C-reactive protein were, however, positively correlated with increasing levels of albuminuria, with the highest levels observed in patients with macroalbuminuria.

In a cohort study of 80 patients followed for 6 months, there was no change in degree of albuminuria from baseline (10). This was attributed to both the short follow-up time in addition to the decision not to exclude patients who had been prescribed either angiotensin-converting enzyme inhibitors or angiotensin receptor blockers, with their well-known anti-albuminuric effects (70). Nevertheless, at baseline and at 6 months, interleukin-6 and C-reactive protein were significantly correlated with elevated urinary albumin excretion.

The receptors for tumor necrosis factor have also been explored as markers of renal injury. In a cross-sectional analysis of 607 patients, the level of soluble tumor necrosis factor receptors 1 and 2 is positively correlated with the presence of diabetic kidney disease (71). Similarly in a study of 83 patients, tumor necrosis factor receptors 1 and 2 were associated with early structural changes seen in diabetic kidney disease such as glomerular basement membrane width and podocyte foot process width (72). Increased risk of end-stage renal disease was correlated with tumor necrosis factor receptors 1 and 2 in 193 American Indian patients followed for a mean of 9.5 years (73).

Although only observational in nature, these studies demonstrate that in patients with type 2 diabetes and diabetic kidney disease, circulating levels of C-reactive protein, fibrinogen, interleukin-6, and tumor necrosis factor are significantly increased. This may suggest that an inflammatory response involving the innate immune system plays a role in the development of albuminuria in these patients.

#### Acute-Phase Proteins and Pro-Inflammatory Cytokines in the Development of Diabetic Kidney Disease in Type 1 Diabetes Mellitus

**Figure 3:**
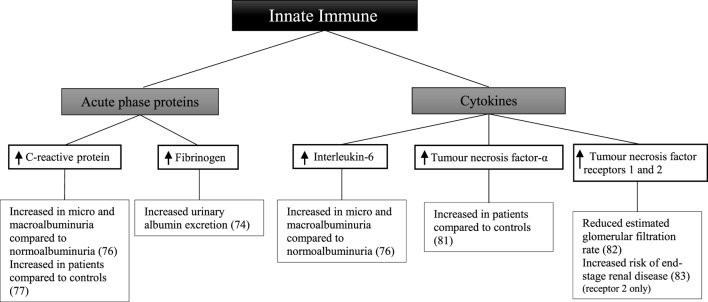
Inflammatory components of the innate immune system and diabetic kidney disease in type 1 diabetes.

There have been a smaller number of studies looking at components of the innate immune system in patients with type 1 diabetes (Figure 3). In 909 patients with type 1 diabetes, there was a difference in the relationship between urinary albumin excretion and circulating fibrinogen in males and females (74). In males, there was a persistent positive correlation at all stages of albuminuria. By contrast, in females, a difference was only seen in those with macroalbuminuria compared with those without. In this study, however, urine and blood to measure urinary albumin excretion and circulating fibrinogen levels were collected within 1 year of each other; hence results may not reflect the true relationship. After 3 years of follow-up, there was no significant difference in C-reactive protein between 49 patients with microalbuminuria and 49 matched controls (75). When analyzing the controls who developed microalbuminuria during follow-up, there was a significant difference in C-reactive protein before and after development of microalbuminuria.

Other studies have shown C-reactive protein and interleukin-6 to be significantly greater in 194 patients with normoalbuminuria compared with 66 healthy controls (76). Furthermore, patients with microalbuminuria and macroalbuminuria had significantly greater levels of C-reactive protein and interleukin-6 than those with normoalbuminuria. Similarly, C-reactive protein was significantly higher in 40 patients compared with 40 matched controls irrespective of albuminuria status (77). In patients with known diabetic kidney disease low-grade inflammation, calculated based on C-reactive protein, interleukin-6, soluble intercellular adhesion molecule-1, and secreted phospholipase A2, was shown to be associated with an increased risk of all-cause mortality after 10 years of follow-up (78). Similarly the risk of the combined cardiovascular endpoint; taking into account cardiovascular-related death, non-fatal event or need for intervention was significantly higher in patients with diabetic kidney disease at baseline. Soluble intercellular adhesion molecule-1, which is associated with vascular wall inflammation (79), has been shown to be significantly associated with the progression from normoalbuminuria or microalbuminuria to macroalbuminuria after a follow-up period of 6 years in an African-American population of 725 patients with type 1 diabetes (80).

A comparison of 44 patients and 24 healthy controls showed increased circulating tumor necrosis factor-α in the patient group (81). Furthermore, tumor necrosis factor-α and tumor necrosis factor receptors 1 and 2 were associated with a lower estimated glomerular filtration rate in 667 patients (82). In a follow-up study of 349 patients with diabetic kidney disease, followed for 5–18 years, baseline tumor necrosis factor receptor 2 was associated with an increased risk of end-stage renal disease (83).

Some studies have examined whether these markers are related to diabetic complications in patients with type 1 diabetes. In 543 patients divided into two groups based on the presence or absence of any complication of type 1 diabetes, circulating levels of C-reactive protein, interleukin-6, and tumor necrosis factor-α were increased in individuals with complications compared with those without (84). Similarly C-reactive protein and fibrinogen were higher in 88 patients without evidence of macrovascular complications compared with 40 controls (85). The patient group was also stratified based on the presence of microvascular complications. When comparing patients with advanced complications to both those without complications and with early-stage complications, a significant difference in the above markers was seen.

Such studies suggest there is an underlying systemic inflammatory process occurring in patients with type 1 diabetes and its complications including diabetic kidney disease. Whether the inflammation occurring in individuals with type 1 diabetes is comparable those with type 2 diabetes is not known and requires further investigation.

#### The Complement Cascade in the Development of Diabetic Kidney Disease

The complement cascade is a highly sophisticated network of immune proteins that are activated in response to invading pathogens or tissue injury. Under normal conditions complement is tightly regulated by a number of fluid-phase and cell surface proteins (86); however, when complement is hyperactivated it drives a severe inflammatory response (87). The complement system comprises over 40 proteins that are present either in soluble form (mostly in blood) or in a bound form at local inflammatory sites and in cell membranes. The soluble proteins normally exist in an inactive form that can be rapidly activated and amplified by a series of tightly regulated sequentially acting proteases. Complement comprises four major pathways that are initiated through antibody/antigen complexes (classical pathway), carbohydrate moieties (lectin pathway), foreign surfaces (alternative pathway), and activated neutral proteases that can cleave C5, especially during inflammatory conditions independent of C3 (extrinsic pathway). All pathways result in the formation of C5a, a major effector molecule which, *via* ligation with its receptor C5aR1, initiates pathology in a number of inflammatory diseases (88).

The kidney appears to be particularly vulnerable to complement-mediated injury (86). Injury may arise from deposition of circulating active complement fragments in glomeruli. Alternatively, local production and activation of complement in the kidney may lead to inflammation. Several renal disorders have been associated with abnormal complement activation (86, 89) including immune complex-mediated glomerulonephritis and tubulointerstitial injury associated with progressive proteinuric diseases (86) in addition to the more recently introduced pathological entity C3 glomerulopathy (89). Excessive complement activation is also involved in two rare but severe kidney diseases that often culminate in end-stage renal failure, membranoproliferative glomerulonephritis type II (dense-deposit disease), and atypical hemolytic uremic syndrome.

It is increasingly appreciated that the complement pathway is activated in diabetic kidney disease (90). Deposition of the membrane attack complex (C5b-9) has been shown in the kidney of patients with diabetic kidney disease (91). A known inhibitor of the membrane attack complex, anti-glycated human CD59-specific antibody, has reduced activity in erythrocytes of diabetic patients (92). This may help to explain the increased membrane attack complex deposition in patients with diabetes (92). In addition to membrane attack complex, deposition in the kidney of diabetic patients with moderate and severe glomerulosclerosis is correlated with medial smooth muscle injury in intrarenal arteries (93).

Serum concentrations of mannose-binding lectin, a key protein involved in the lectin pathway, are increased in patients with diabetic kidney disease (94) and are predictive of the subsequent development of albuminuria (95). Ostergaard et al. found that serum H-ficolin, an activator of the lectin pathway, was associated with an increased risk of future progression to microalbuminuria in patients with newly diagnosed type 1 diabetes (96). In a mouse model of type 1 diabetes, circulating levels of mannan-binding lectin significantly increased after induction (97). Similarly in humans, mannan-binding lectin is significantly higher in diabetic patients compared with healthy controls (98). Furthermore, mannan-binding lectin positively correlates with urinary albumin excretion in patients with type 1 diabetes (98–101) as well as type 2 diabetes (102). Streptozotocin-induced diabetic mice with a genetic deletion in mannose-binding lectin had significantly lower rates of urinary albumin excretion compared with diabetic wild-type mice (103).

During a median of 5.8 years follow-up, baseline mannose-binding lectin was associated with increased urinary albumin excretion in patients with type 1 diabetes (104). Mannose-binding lectin was also significantly associated with progression from macroalbuminuria to end-stage renal disease (104). Similarly after a median of 18 years follow-up of newly diagnosed type 1 diabetic patients’ mannose-binding lectin measured after 3 years was associated with the development of microalbuminuria and macroalbuminuria (95). After 15 years of follow-up, the mortality of patients with type 2 diabetes was found to be significantly associated with baseline mannose-binding lectin (105). A recent study using genome-wide transcriptome analysis has shown upregulation of the complement pathway in micro-dissected human renal glomerular and tubule samples from patients with diabetic kidney disease (106). Although these studies indicate an association between activation of the complement pathway and diabetic kidney disease, the role of specific complement factors in mediating cellular injury in the development and progression of diabetic kidney disease has not yet been determined. It remains to be determined if complement proteins are useful markers for chronic kidney disease progression.

### Role of Leukocytes in Diabetes Mellitus and Diabetic Kidney Disease

**Figure 4:**
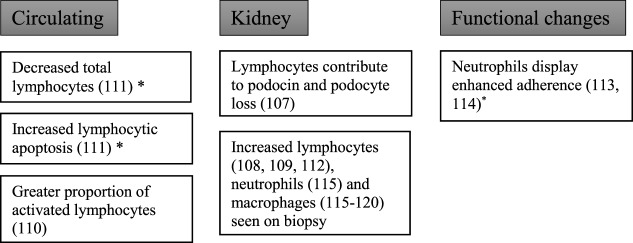
Changes to leukocytes in diabetic kidney disease.

Studies at the cellular level have demonstrated that not only do leukocytes infiltrate the kidneys in patients with diabetic kidney disease but that functional changes in these cells also occur (Figure 4).

#### Lymphocytes

To explore the role of lymphocytes in the development of diabetic nephropathy, Lim et al. studied recombination activating gene-1-deficient mice made diabetic with streptozotocin (107). Recombination activating gene-1-deficient mice lack mature T and B cells and hence allowing the study of the development of diabetic kidney disease in absence of lymphocytes. Diabetic recombination activating gene-1-deficient mice showed a reduction in albuminuria compared with diabetic wild-type mice in addition to preserved podocytes and reduced glomerular macrophage accumulation. These features suggest that lymphocytes play a role in the development of albuminuria. However, it should be noted that both groups showed equal levels of decline in creatinine clearance and other characteristic histological features of diabetic kidney disease.

Another study using non-obese diabetic mice (NOD mice, a model of autoimmune diabetes) showed that diabetic mice had T cell and B cell infiltration into the glomeruli, as well as CD11c+ dendritic cells (108). There was also deposition of glomerular IgG and complement C3. A similar study showed at both 1 and 8 months post diabetes induction (STZ) in rats, there was an increased infiltration of CD4+ T cells, CD8+ T cells, and macrophages into the glomeruli (109). The change in CD4+ cells appeared to be more significant at 1 month, whereas CD8+ T cells become more significant at later stages of the disease when tissue loss was evident.

Clinical studies have also shown lymphocyte activation in diabetes. In patients with type 2 diabetes and proteinuria, there was no difference in absolute numbers of circulating lymphocytes compared with age-, sex-, and duration-of-diabetes-matched controls with type 2 diabetes and without proteinuria (110). There was, however, a higher proportion of activated lymphocytes, as measured by an antihuman leukocyte antigen D-related antigen, in diabetic patients with non-nephrotic range proteinuria compared with non-proteinuric diabetic controls.

Otton et al. performed a study investigating apoptosis of lymphocytes obtained from diabetic subjects or from rats with alloxan-induced diabetes (111). Lymphocytes harvested from rat mesenteric lymph nodes or human circulating lymphocytes showed significantly higher rates of fragmented DNA, chromatin condensation, and blebbing. The high occurrence of apoptosis in lymphocytes was accompanied by a reduced number of blood-circulating lymphocytes in diabetic patients. Interestingly, in rats with insulin therapy, the proportion of lymphocytes with fragmented DNA was decreased.

Furthermore, Moon et al. have found increased infiltration of interstitial CD4+ and CD8+ T cells in 20 weeks diabetic mice kidney when compared with non-diabetic controls (112). Using flow cytometry, intrarenal CD4+ and CD8+ T cells were also significantly increased in proteinuric diabetic mice. However, the population of T and B cells in diabetic spleen was not changed, indicating the changes in lymphocytes in diabetes occurred predominantly in the kidney and not within the circulation. More importantly, in the same study, there was a significant increase in the infiltration of renal interstitial T (CD4+, CD8+) and B cells (CD20+) in type 2 diabetic human kidney, and the increase in CD4+ T cells and CD20+ B cell correlated with the amount of proteinuria in these patients (112).

#### Neutrophils

In 11 patients with type 1 diabetes and albuminuria, there was a significant downregulation of the adhesion molecule CD11b on neutrophils following activation by phorbol 12-myristate 13-acetate (113). The pathways controlling granule exocytosis in neutrophils from diabetic patients were shown to be abnormal. In type 2 diabetes, neutrophils display enhanced adherence to fetal-calf serum coated surfaces (114). In 22 renal biopsies from patients with biopsy proven diabetic kidney disease, there was increased infiltration of neutrophils and macrophages into interstitial, peritubular, and capillary regions (115). Furthermore, the number of interstitial and peritubular renal polymorphic neutrophils in diabetic nephropathy was proportional to estimated glomerular filtration rate at the time of the biopsy. The study did not report whether patients had type 1 diabetes or type 2 diabetes.

#### Macrophages

Kidney macrophage accumulation is associated with the progression of type 2 diabetic nephropathy. Chow et al. performed a time course of renal disease progression and kidney macrophage infiltration in the db/db mouse model, which exhibits obesity and type 2 diabetes (116). In db/db mice at 8 months of age, there was an increased macrophage infiltration in glomerular and interstitial regions of the kidney in mice which developed hyperglycemia compared with those that did not. Macrophage accumulation and activation in db/db mice correlated with HbA_1c_, glomerular and tubular damage, albuminuria, elevated plasma creatinine, renal fibrosis, and kidney expression of macrophage chemokines. In another study looking at 20 patients with diabetic kidney disease, macrophage accumulation in both glomerular and interstitial regions of renal biopsies was demonstrated (117). However, only infiltration into the interstitium was found to be correlated with progression of disease. Total macrophage count was shown to correlate with renal impairment but not albuminuria. In renal biopsies of patients with histologically proven diabetic kidney disease, compared with controls with no diabetic kidney disease, the presence of glomerular anti-inflammatory CD163+ macrophages were significantly associated with the presence of glomerulosclerosis, interstitial fibrosis, and tubular atrophy (118). Similarly both estimated glomerular filtration rate and level of albuminuria were associated with the presence of interstitial CD68+ macrophages.

In streptozotocin-induced diabetic mice, there was increased macrophage infiltration in both glomeruli and the interstitium when compared with controls (119). In the same study, genetic knockout of macrophage scavenger receptor A, which results in a reduction of macrophage infiltration into the kidneys, afforded renoprotection from STZ diabetes as shown by decreased albuminuria, glomerular hypertrophy, and renal fibrosis. It has been shown that this infiltration occurs early in diabetic kidney disease development, with macrophage infiltration being seen by immunohistochemical labeling 8 days after diabetes induction in streptozotocin-induced diabetic rats (120). However, streptozotocin may have acute effects. Nonetheless, in one study using another model of type 1 diabetes, the spontaneously diabetic Ins2^Akita^ mouse, increased accumulation of macrophages was also observed in the glomeruli of diabetic Ins2^Akita^ mice after 14–15 weeks of diabetes (121), thus suggesting that the recruitment of macrophages into the diabetic kidneys is not an exclusive feature of streptozotocin in animal models of type 1 diabetes.

A key mediator in the infiltration of macrophages is monocyte chemoattractant protein-1 (122). In response to pro-inflammatory cytokines interleukin-1, tumor necrosis factor-α, and interferon-γ, monocyte chemoattractant protein-1 is released from kidney tubular cells, smooth muscle cells, mesangial cells, and podocytes (122). In addition to macrophage accumulation, monocyte chemoattractant protein-1 is thought to be indirectly involved in the recruitment of neutrophils through an intermediate mediator leukotriene B4 (123). Monocyte chemoattractant protein-1 deficient diabetic mice do not develop albuminuria and are protected from an increase in plasma creatinine (124). The P2X7 receptor is expressed on macrophages and mediates pro-inflammatory signaling pathways (125). In human mesangial cells exposed to a high glucose environment, P2X7 receptors have increased release of monocyte chemoattractant protein-1 (125). Furthermore, mice with a knockdown of the P2X7 receptor had reduced glomerular macrophage recruitment and collagen IV deposition (125).

The use of NOX-E36 (empaticap pegol) which binds and inhibits MCP-1 resulted in a reduction of albumin–creatinine ratio in patients with type 2 diabetes (126). Furthermore, treatment with CCX140-B, an antagonist to C–C chemokine receptor 2 which monocyte chemoattractant protein-1 binds to, resulted in a reduction in albumin–creatinine ratio (127), glomerular hypertrophy, and increased podocyte density in experimental diabetes (127). Treatment with CCX140-B in patients with type 2 diabetes and nephropathy resulted in a reduced albumin–creatinine ratio (5).

### Emergence of Neutrophil–Lymphocyte Ratio as a Potential Marker of Diabetic Kidney Disease

Both neutrophils and lymphocytes have been shown to have altered function in patients with type 1 diabetes and type 2 diabetes. Neutrophils are positively correlated with urinary albumin excretion in patients with type 2 diabetes while lymphocytes are negatively correlated (53).

**Figure 5:**
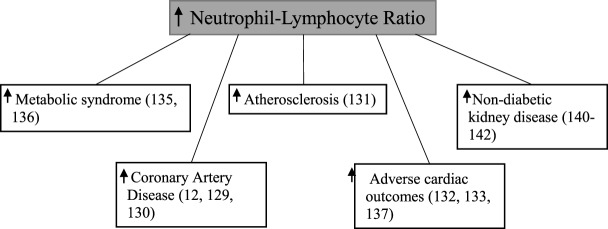
Elevated neutrophil–lymphocyte ratio and various disease states.

Neutrophil–lymphocyte ratio, calculated by dividing the total neutrophil count by the total lymphocyte count (128), has been shown to be associated with coronary artery disease (7, 129, 130), atherosclerosis formation (131), adverse cardiac outcomes (132–134), and the metabolic syndrome (135, 136) (Figure 5). The neutrophil–lymphocyte ratio is a cheap, commonly available (137), and stable marker which provides better predictive value than individual leukocyte counts (138, 139).

There have been a small number of studies relating neutrophil–lymphocyte ratio to non-diabetic kidney disease. Sixty one dialysis patients with higher neutrophil–lymphocyte ratio were found to also have significantly elevated levels of tumor necrosis factor-α (140). Neutrophil–lymphocyte ratio has also been demonstrated to be significantly higher in patients with chronic kidney disease when compared with healthy controls (141). Finally in 105 patients with stage 4 kidney disease, a higher baseline neutrophil–lymphocyte ratio was significantly associated with a rapid decline in estimated glomerular filtration rate, which was defined as >5 ml/min/1.73 m^2^/year (142).

#### Use of Neutrophil–Lymphocyte Ratio in Diabetes Mellitus

**Figure 6:**
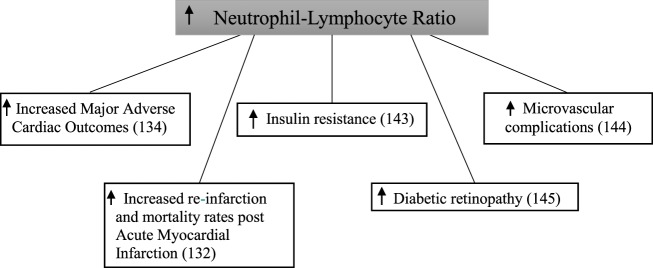
Increased neutrophil–lymphocyte ratio and complications in diabetes mellitus.

A relationship between neutrophil–lymphocyte ratio and the metabolic syndrome has been established (122, 123). Furthermore, there is a common inflammatory pathway observed between cardiovascular disease and type 2 diabetes (30). Several studies have examined the relationship between neutrophil–lymphocyte ratio and complications of type 2 diabetes (Figure 6).

Baseline neutrophil–lymphocyte ratio was significant higher in patients with type 2 diabetes compared with those without in a study following 2,559 patients for 1 year after being admitted to hospital with an acute myocardial infarction (132). After follow-up, the composite endpoint of re-infarction and mortality increased in a step-wise manner across quartiles of neutrophil–lymphocyte ratio in patients with type 2 diabetes. This did not occur in patients without type 2 diabetes. In a retrospective cohort study of 338 patients with type 2 diabetes, those in the highest tertile of baseline neutrophil–lymphocyte ratio had significantly increased rates of major adverse cardiac events and mortality compared with those in the lowest tertile (134).

In a cross-sectional analysis of 586 patients, neutrophil–lymphocyte ratio was significantly higher in patients with type 2 diabetes when compared with those with normal glucose tolerance (143). This was similarly shown when comparing patients with type 2 diabetes to those with impaired glucose tolerance. Furthermore, patients with impaired glucose tolerance had a significantly higher neutrophil–lymphocyte ratio than those with normal glucose tolerance. Neutrophil–lymphocyte ratio was significantly higher in 146 patients with type 2 diabetes with microvascular complications compared with 97 patients without microvascular complications (144). Patients with microvascular complications also had a significantly higher neutrophil–lymphocyte ratio when compared with 218 healthy controls. Interestingly, no difference was seen between the control group and the patients with type 2 diabetes without microvascular complications. In this study, diabetic kidney disease was diagnosed based on urinary albumin excretion alone. In 58 type 2 diabetic patients, neutrophil–lymphocyte ratio was significantly higher when compared with 52 controls (145). In addition to this, neutrophil–lymphocyte ratio was also significantly higher in participants who also had diabetic retinopathy.

These studies demonstrate that inflammation, as measured by neutrophil–lymphocyte ratio, is associated with both macrovascular and microvascular diabetic complications.

#### Use of Neutrophil–Lymphocyte Ratio in Diabetic Kidney Disease

**Figure 7:**
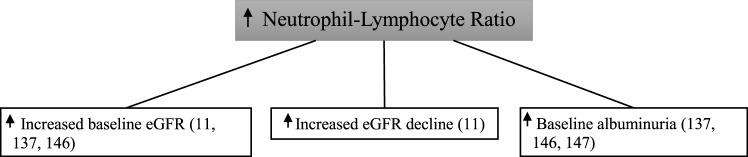
Increased neutrophil–lymphocyte ratio and diabetic kidney disease.

To date, only a handful of studies have looked at the association between neutrophil–lymphocyte ratio and diabetic kidney disease (Figure 7).

In 338 type 2 diabetic patients followed for 3 years, those with a higher baseline neutrophil–lymphocyte ratio were found to have increased rates of the primary endpoint of >12 ml/min loss in estimated glomerular filtration rate over the 4-year study period with the last estimated glomerular filtration rate being <60 ml/min (11). Similarly, a high baseline neutrophil–lymphocyte ratio correlated with a low baseline estimated glomerular filtration rate in this study. In 114 patients with type 2 diabetes, neutrophil–lymphocyte ratio was significantly higher in those with diabetic kidney disease compared with those without—with the presence of diabetic kidney disease being defined on the basis of both albuminuria and estimated glomerular filtration rate criteria (137). In a multiple logistic regression analysis, neutrophil–lymphocyte ratio was independently predictive of diabetic kidney disease. In this study, there was no association seen between neutrophil–lymphocyte ratio and diabetic retinopathy.

In a cross-sectional analysis of 200 patients with type 2 diabetes, elevated neutrophil–lymphocyte ratio was significantly associated with microalbuminuria, macroalbuminuria, and reduced estimated glomerular filtration rate (146). Neutrophil–lymphocyte ratio was significantly higher in 115 patients with type 2 diabetes and early-stage diabetic kidney disease when compared with 138 type 2 diabetics with no diabetic kidney disease (147). However, early-stage diabetic kidney disease was defined as urinary albumin excretion within the microalbuminuria range only. Furthermore, patients with type 2 diabetes who had declining estimated glomerular filtration rate without evidence of albuminuria were excluded.

These studies demonstrate that changes in both the innate immune system by way of the total neutrophil count and the adaptive immune system in terms of the total lymphocyte count are associated with the development of diabetic kidney disease.

## Conclusion

Although diagnosis of diabetic kidney disease is well established, there is ongoing research to identify cheap and accessible ways to detect disease at an earlier stage and thus facilitate early intervention. It is hoped that through such research, reliable ways of predicting future risk may also emerge. Given the link between diabetes and inflammation, the use of common blood tests including the white cell count which is used to calculate the neutrophil–lymphocyte ratio may help provide this early detection.

Previous studies have traditionally used urinary albumin excretion as an indicator of the presence of diabetic kidney disease, along with estimates of glomerular filtration rate. It is important to recognize the limitations of using urinary albumin excretion alone in patients with type 2 diabetes to determine presence of diabetic kidney disease.

Observational studies have shown positive associations between inflammatory mediators, such as C-reactive protein, and the development of diabetic kidney disease. This has been demonstrated in both patients with type 1 and type 2 diabetes and suggests that activity of the innate immune system is implicated in this development. Furthermore, diabetic kidney disease is positively associated with neutrophils and negatively associated with lymphocytes. This suggests both the innate and adaptive immune systems play a role in the pathogenesis of diabetic kidney disease. This is further supported by the association between diabetic kidney disease and the neutrophil–lymphocyte ratio. Due to the lack of specificity of the neutrophil–lymphocyte ratio, its ability to detect diabetic kidney disease is not superior to that of albuminuria. However, it does provide important insight into the mechanisms that drive diabetic kidney disease development.

Further research is required to understand in detail how this interaction occurs as it may lead to the development of interventions. Treatments that are able to stop the key mediators of inflammation in these patients could not only reduce rates of diabetic kidney disease but also help to stop progression to end-stage renal disease. Renoprotective effects of anti-inflammatory agents which block the effects of monocyte chemoattractant protein-1 reducing albuminuria have recently been demonstrated. Other medications that affect inflammation are being developed to target diabetic kidney disease.

However, longitudinal studies with long-term follow-up periods are required to determine the exact role of inflammation and key mediators of the immune system in the development of diabetic kidney disease. Furthermore, treatments which block known inflammatory mediators in the pathogenesis of diabetic nephropathy need further research to determine if these are viable targets for treatment in patients with diabetes.

## Author Contributions

LW—primary author of review. LAW—contributed to background research and references. GJ and RM—co-supervisor. J-mS and MC—helped with editing manuscript. ST and MTC—primary author of complement subsection. EE—main supervisor of the manuscript- supervised the research, and edited the manuscript.

## Conflict of Interest Statement

The authors declare that the research was conducted in the absence of any commercial or financial relationships that could be construed as a potential conflict of interest. The reviewer FP and handling Editor declared their shared affiliation.
